# Benefits of Judo Practice for Individuals with Neurodevelopmental Disorders: A Systematic Literature Review

**DOI:** 10.3390/sports12070182

**Published:** 2024-06-27

**Authors:** Gaston Descamps, Maria João Campos, Terry Rizzo, Viktorija Pečnikar Oblak, Alain Guy Massart

**Affiliations:** 1 Faculty of Sports and Physical Education, University of Coimbra, 3040-248 Coimbra, Portugal; mjcampos@fcdef.uc.pt (M.J.C.); alainmassart@fcdef.uc.pt (A.G.M.); 2 Research Unit for Sport and Physical Activity (CIDAF), University of Coimbra, 3040-248 Coimbra, Portugal; 3 Department of Kinesiology, California State University, San Bernardino, CA 92407, USA; trizzo@csusb.edu; 4 Institute of Social Welfare of the Republic of Slovenia, 1000 Ljubljana, Slovenia; viktorija.pecnikar-oblak@irssv.si

**Keywords:** combat sports, autism spectrum disorder, intellectual developmental disorders, attention-deficit/hyperactivity disorder, systematic review

## Abstract

This systematic literature review evaluates the benefits of judo practice designed for individuals with neurodevelopmental disorders (NDDs), including autism spectrum disorder (ASD), Intellectual Developmental Disorders (IDDs), and attention-deficit/hyperactivity disorder (ADHD). This review adheres to the PRISMA 2020 guidelines, focusing on the physical, social, emotional, and cognitive benefits of judo. A comprehensive search across databases, such as PubMed, Google Scholar, ResearchGate, B-On, and Scopus, was conducted, and relevant studies were selected based on explicit inclusion and exclusion criteria. Sixteen intervention studies were included, which contributed to a detailed understanding of the impact of judo. The results indicated significant benefits in terms of physical activity, social interactions, emotional well-being, and cognitive functions among participants. A synthesis of results is presented, showing the overall positive effect of judo practice. This review highlights the potential of judo as supportive therapy for individuals with NDDs, advocating its inclusion in therapeutic and educational settings. Limitations due to study heterogeneity and the need for more randomized controlled trials are also discussed.

## 1. Introduction

The significance of engaging in sports and physical activities is universally acknowledged, extending to populations with disabilities [1]. A growing body of academic literature underscores the comprehensive physical, psychological, and social benefits of regular participation in sports, exercise, and physical activities for individuals with disabilities [2]. Neurodevelopmental disorders, such as autism spectrum disorder (ASD), attention-deficit/hyperactivity disorder (ADHD), intellectual developmental disorder (IDD), and other related conditions, manifest early in life and involve significant neuropsychiatric challenges. The prevalence and societal awareness of these conditions have increased, reflecting a growing recognition of their impact on public health [3,4]. Judo, established by Jigoro Kano in 1882, is not only a sport but also a philosophy that emphasizes the optimal use of energy (“Seiryoku zen.yô”) and mutual prosperity (“Jita Kyoei”). These principles advocate for judo’s personal and societal benefits, facilitating physical robustness and promoting psychological and social well-being [5]. However, despite its widespread adoption and philosophical depth, the application of judo in the context of disability and inclusion, particularly for individuals with NDDs, remains relatively underexplored at an academic level. In recent years, judo for individuals with disabilities has gained recognition for its diverse applications, spanning recreational pursuits with socialization goals, competitive engagement through various federations and organizations, and therapeutic interventions promoting health and rehabilitation, as highlighted in the systematic review for populations with IDD from Oblak et al. [6]. The practice of judo is being adapted to the public with NDDs with the main aim of ensuring the safety of participants. For example, to protect the neck, every movement or technique that applies pressure on the neck is avoided during training and forbidden during competitions, the same goes for chokes, arm-locks, and sacrifice techniques; all the prohibited actions are detailed in the European Judo Union rules for adapted judo [7]. A very recent study showed that the injury rate for participants with IDD is similar to the one for participants in mainstream judo due to these adaptations, indicating that the practice of judo is safe for a population with NDDs [8]. This review will analyze the current literature on judo practice for a population with NDDs, focusing on its benefits and how the practice is structured to accommodate individuals with NDDs to contribute to their development across various dimensions. This systematic review expands upon the findings of Oblak et al. [6] by incorporating recent studies and studies including more types of NDDs than IDD, thereby providing a comprehensive synthesis of the available literature on the impact of judo on individuals with NDDs. By examining existing research and scientific papers, we aim to shed light on the benefits of judo in answering the different challenges that participants with NDDs have in their lifetime development at all physical, social, emotional, and cognitive levels.

## 2. Method

This systematic review adheres to the PRISMA [9] guidelines to investigate the effects of judo practice on individuals with neurodevelopmental disorders (NDDs). The review process is organized into distinct sections as follows:

### 2.1. Protocol

The protocol for this review was established prior to conducting this study, ensuring alignment with the PRISMA [9] guidelines to promote transparency and reproducibility. The study protocol was registered in the Open Science Framework (OSF) repository with the DOI 10.17605/OSF.IO/VY5U4 to ensure the accessibility of the methodology and enhance the integrity of the review process.

### 2.2. Eligibility Criteria

Peer-reviewed articles, dissertations, randomized controlled trials (RCTs), quasi-experimental studies, cross-sectional analyses, and observational studies were included in the review. Additionally, non-journal publications such as student theses were considered if they met peer-reviewed standards. Eligible studies involved individuals with NDDs engaging in judo published within the last 30 years in English, French, Portuguese, or Spanish. This timeframe and linguistic inclusivity aimed to capture a comprehensive scope of research developments.

### 2.3. Information Sources

The primary information sources included major databases such as PubMed, Google Scholar, ResearchGate, B-On, and SCOPUS. These were supplemented by reference lists and relevant organizational websites to ensure a thorough search.

### 2.4. Search

The searches were conducted on 1 September 2023. Our search strategy utilized a comprehensive set of keywords: “Judo” AND (“Neurodevelopmental Disorder” OR “Intellectual Developmental Disorders” OR “Intellectual Disability” OR “Autism Spectrum Disorder” OR “ADHD” OR “Neurodevelopmental Motor Disorders” OR “Tic Disorders” OR “Specific Learning Disorders”). Filters were applied to limit results to the specified publication window and languages, with all search strings meticulously documented to aid replication.

### 2.5. Study Selection

Study selection was carried out meticulously. The first author initially screened each record, followed by a review by the supervising author and three co-authors. This ensured a rigorous selection process. No automation tools were used in the screening process.

### 2.6. Risk of Bias

The risk of bias and standard systematic evaluation was assessed using appropriate tools: the STROBE checklist [10] for observational studies, the TREND checklist [11] for quasi-experimental studies, and the CONSORT checklist [12] for RCTs. Each study was assessed by the first author. A threshold of 80% was established for inclusion, and any articles falling below this threshold were excluded from the analysis.

### 2.7. Data Collection Process

Data collection focused on all outcomes relevant to the effects of judo on NDDs. The results of the included studies were synthesized using the PICOTS framework [13].

## 3. Results

**Figure 1:**
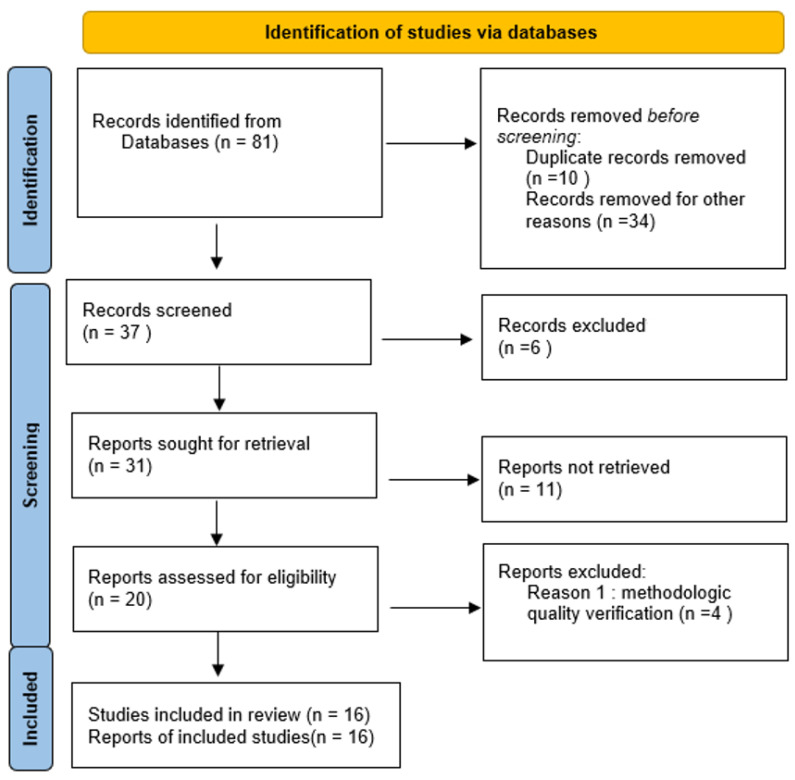
Flow diagram (PRISMA 2020) for new systematic reviews which included searches from database.

The initial research identified a total of 81 articles on the baselines of (Pub Med (n = 25), Research Gate (n = 15), Google Scholar (n = 18), Bo-on (n = 8), and SCOPUS (n = 15)). After the importation of all the references in Mendeley, we automatically eliminated the articles in double (10); we manually eliminated 34 articles based on the title and the abstract to obtain a result of 37; 6 more articles were eliminated because they were not intervention studies. In the end, we reviewed these 31 articles, which resulted in the elimination of 11 more because they were not about our theme and did not correspond to the specific aim of our review, which resulted in a total of 16 articles included in this review (Figure 1).

Table 1 presents the results of the included studies using the PICOTS framework [13]; they encompass both quantitative and qualitative outcomes. 

### 3.1. Design and Setting

Of the 13 studies that focus on youth with ASD, regarding program duration, the shortest one, from Tomey [14], is five weeks, and the longest are 6 months, including the studies by Morales et al. [24], Couto [20], and Pierantozzi et al. [25]. The most common duration among the studies is 8 weeks, which includes studies by Garcia et al. [18], Rivera et al. [16], Burrell [15], Rivera et al. [19], Renziehausen et al. [17], and Morales et al. [21]. Regarding the frequency of sessions, nine studies were conducted with weekly sessions, while three studies, including those of Tomey [14], Renziehausen et al. [17], and Garcia et al. [22], had bi-weekly sessions. The duration of the sessions varied from 45 min to 90 min, with the longest sessions found in the studies by Morales et al. [24] and Pierantozzi et al. [25]. Most of the studies specified the presence of more than one instructor during the sessions. In terms of the setting, 12 out of the 13 studies were specifically designed only for youth with ASD. The study by Garcia et al. [23] compared a group of youth with ASD with a group of youth with ASD and their parents together. Only one study, Tomey [14], had an intervention program made in an inclusive setting and studied the benefits of this setting.

Each of the two studies with participants with IDD differs in terms of duration, frequency, number of participants, and information about the instructors involved. Boguszewski et al. [28] had the longest duration of 12 months. The number of participants varied, with Boguszewski et al. [28] having 35 participants in the experimental group and Zamanillo [27] involving 17 adults with IDD. 

The study conducted by Ludyga et al. [29] lasted for 3 months and involved weekly sessions lasting 120 min each. In the study, 28 children with ADHD were assigned to the experimental group, while another 28 children were placed in the control group. 

### 3.2. Sample Characteristics

Tomey [14] investigated five children with ASD and five typically-developed children aged 8–11 years; as already mentioned, it is the only study made in an inclusive environment. Burrell [15] studied 20 youth with ASD aged 8–17 years, and Rivera et al. [16] and Rivera et al. [19] each analyzed 25 youth with ASD within the same age range. Renziehausen [17] focused on 20 youths with ASD spanning from children to adolescents. Garcia et al. [18] included 14 youth with ASD aged 8–17 years, and Garcia et al. [22] looked at nine adolescents with ASD, predominantly male, averaging around 16.87 years. Garcia et al. [23] explored the dynamics involving nine youths with ASD, their parents, and youth without parental involvement. Morales et al. [21] and Morales et al. [24] examined youth with ASD aged 9–13 years and 11.07 years on average, respectively, both with specific IQ ranges (60–70 and 55–70). Couto [20] focused on 10 youth with ASD aged 6–14 years. Pierantozzi et al. [25] and Lockard et al. [26] provided insights into broader age ranges and developmental conditions, including various disabilities among youth aged 4–23 years. Concerning the IDD population, Zamanillo [27] studied 17 adults aged 18–49 years, and Boguszewski et al. [28] focused on 73 children, mostly with IDD, averaging 11.7 years. Ludyga et al. [29] contributed findings on 57 children with ADHD aged 8–12 years.

### 3.3. Intervention Description

The programs constructed for participants with ASD consistently emphasize the teaching of judo techniques and principles, with a strong focus on safety and mindfulness. Those aspects exist as well in mainstream judo programs. Nonetheless, the safety component is particularly emphasized in adapted judo programs as well as the mindfulness component, which is sometimes forgotten in mainstream judo programs. For example, studies conducted by Burrell [15], Garcia et al. [23], Garcia et al. [22], and Rivera [16] all emphasize the importance of safe falling techniques to ensure participant safety during practice sessions. Most interventions include a warm-up phase, a main exercise/technical segment, and a cool-down period. The warm-up phase typically involves activities like jogging, stretching, and tumbling, as described in the study by Garcia et al. [18]. As mentioned, several studies (eg: Burrell [15]; Garcia et al. [23], Rivera et al. [19], Rivera [16], and Garcia et al. [18]) incorporate mindfulness and reflection activities into the closing minutes of each session, allowing participants to reflect on their practice and cultivate a sense of presence and awareness. Including mindfulness in the sessions demonstrates a shared understanding of its potential benefits for individuals with ASD, such as enhancing focus and self-regulation skills [30,31], but also for other types of neurodevelopmental disorders, like ADHD ([32,33]. The teaching of the foundational movements and partner-centered exercises, as seen in studies by Garcia et al. [23] and Morales et al. [21], were part of most programs. They also incorporated ground control techniques and throws, progressively adding complexity to the practice, as mentioned in studies by Morales et al. [21] and Morales et al. [24]. Moreover, adaptations specifically tailored for individuals with ASD are evident in certain studies. For instance, Morales et al. [24] emphasize the need for clear and repetitive instructions and monitoring and redirecting spontaneous behavior changes to adapt to specific learning needs. The instructional approaches employed in the studies are also highlighted. Some interventions, like those of Morales et al. [21] and Morales et al. [24], emphasize imitation and guided modeling, allowing participants to progress at their own pace and ensuring a comfortable learning environment. Burrell [15] and Pierantozzi et al. [25] adopt a gradual progression approach, consolidating concepts before introducing more complex material. These instructional approaches indicate the researchers’ recognition of the diverse learning styles and needs of individuals with ASD and their efforts to accommodate them. With didactical approaches, adaptations for individuals with ASD, and specific content covered, they underscore the flexibility and adaptability of judo as an intervention and emphasize the need for tailored approaches to meet the unique needs of individuals with ASD. 

Regarding programs constructed for participants with IDD, Zamanillo [27] highlighted broader objectives of judo programs, encompassing physical and social development. The goals included learning skills for navigating the sports and social environments, developing moral values and social responsibility, and fostering self-esteem and emotional intelligence. Those programs aimed to promote physical activity, healthy lifestyle habits, and a positive self-image. Technical–tactical aspects and values of combat sports were covered, along with specific warm-up exercises and various combat techniques. Unfortunately, the study of Boguszewski et al. [28] did not provide details about the structure of the program or session.

For the program dedicated to participants with ADHD, the study by Ludyga et al. [29] aimed to train novice judo practitioners to prepare for their initial belt examination. The training focused on teaching fundamental judo techniques encompassing offensive moves, defensive techniques, and injury prevention strategies. While the primary emphasis was on technique acquisition and improvement, the sessions also incorporated enjoyable and age-appropriate exercises to enhance physical fitness. These two components were integrated and practiced together during “randori” (free sparring) sessions.

### 3.4. Assessment Measures

Quantitative surveys and psychometric tests were foundational in many of the studies. For example, the Physical Activity Self-Efficacy and Physical Activity Enjoyment scales used in Tomey [14], and Burrell [15] measured changes in psychosocial factors through paired samples *t*-tests. Behavioral assessments were another critical component, with tools like the Aberrant Behavior Checklist (ABC) and the Gilliam Autism Rating Scale (GARS) frequently applied to evaluate behavioral symptoms and autism severity, as seen in the works of Rivera et al. [19] and Morales et al. [21].

Physiological measures added a layer of scientific rigor to the studies, with devices like Actigraph accelerometers monitoring physical activity and sleep patterns in Rivera [16], and salivary cortisol levels assessing stress responses in Renziehausen et al. [17]. Objective physical measures also played a crucial role in quantifying physical activity levels and sedentary behavior through statistical methods, as in Garcia et al. [18].

The inclusion of semi-structured interviews and focus groups provided qualitative depth, offering insights from parents, school staff, and participants themselves, enriching the data and highlighting the nuanced impacts of the judo programs. These were particularly evident in the studies of Tomey [14] and Morales et al. [21].

Motor skills and physical fitness were assessed using the Test of Gross Motor Development (TGMD-3) and the ALPHA-fitness battery in studies like Morales et al. [24] and Pierantozzi et al. [25], evaluating improvements in motor skills and physical health.

Adapting to remote delivery was uniquely addressed during the COVID-19 pandemic, with studies like that of Garcia et al. [22] exploring the feasibility and acceptability of remote judo sessions, assessing participant satisfaction, and assessing the practicality of virtual engagement.

Finally, comparative and correlational analyses enriched the understanding of the data, linking attendance to behavioral improvements and psychosocial benefits, as demonstrated in Burrell [15] and Rivera [16].

### 3.5. Category of Outcomes

Table 2 presents a synthesis of the outcomes and their categories (physical, social, emotional, and cognitive) and specifies whether they are quantitative or qualitative results.

## 4. Discussion

This systematic review extensively examines the benefits of judo for individuals with neurodevelopmental disorders (NDDs), with a specific focus on its potential impacts across physical, social, emotional, and cognitive dimensions. While the reviewed studies predominantly center on ASD, ADHD, and IDD, this discussion aims to interpret these findings comprehensively, addressing limitations, identifying gaps in existing research, and suggesting future research directions.

### 4.1. Interpretation of Findings

A consistent theme across the studies is the enhancement of physical activity levels, motor skills, and overall physical health. For instance, the studies by Garcia et al. [18] and Morales et al. [24] document increases in moderate to vigorous physical activity (MVPA) and improvements in motor skills, respectively. These findings are particularly important given that physical activity can often be lower in individuals with NDDs due to various barriers such as motor deficits, social skills limitations, and the lack of appropriate opportunities [34]. Judo, with its structured nature and emphasis on physical engagement through various techniques, appears to successfully address these barriers, enhancing physical fitness and providing a gateway to increase general physical activity [35].

Several studies noted social improvements, increasing social skills, communication, and emotional responses during the program [20,26]. These outcomes likely stem from judo’s intrinsic values woven into its training and practice [36]. The structured interaction within judo can serve as a safe space for practicing social interactions and receiving immediate feedback in a supportive environment [14], which is crucial for individuals with NDDs who may struggle with complex social settings [37].

Emotionally, judo seems to offer a constructive outlet for stress and has been associated with improved self-esteem and psychological well-being [17,27]. The framework of Jigoro Kano’s Kodokan Judo system enhances judo’s benefits in developing emotional intelligence, self-control, and adherence to society [38].

Cognitive improvements, particularly in areas related to executive functioning and memory, were noted in studies such as that by Ludyga et al. [29], which reported enhancements in working memory processes in children with ADHD. This aligns with broader research suggesting that physical exercise can have a positive impact on cognitive functions, likely mediated by neurophysiological changes such as increased cerebral blood flow and neurotrophic factors, which are crucial for brain health and plasticity [39].

### 4.2. Gaps in the Literature

One notable gap is the predominant focus on ASD, with limited research exploring the impact of judo on other NDDs. Additionally, the lack of randomized controlled trials (RCTs) in this domain limits the ability to robustly establish causality between judo interventions and observed outcomes. Such gaps signify a critical need for expansive research that includes diverse NDD populations and employs rigorous methodologies to validate the findings reported by initial studies. Another gap is the scarcity of studies conducted in an inclusive setting.

### 4.3. Limitations 

This review faces limitations due to the heterogeneity of the studies in terms of settings, intervention durations, session frequencies, and outcome measures. The variability in these factors makes it challenging to draw generalized conclusions about the effectiveness of judo practice. 

### 4.4. Implications and Future Directions

Judo’s core principles, such as mutual prosperity and optimal use of energy, naturally support inclusive practices, suggesting potential benefits for a wide range of participants. Future research should focus on exploring the effects of judo within diverse and inclusive settings to better understand its broad applicability and benefits. There is an increasing emphasis on the necessity of inclusivity in physical activity, education, and sports [40]. The study of Colocon et al. [41], which aligns with the UNESCO-backed Sustainable Development Goal 4 [42], advocates for inclusive and high-quality education for all individuals, recognizing the importance of individualized learning that accommodates diverse abilities, attributes, and aspirations. Sit et al. [43] assert that physical education and physical activities are pivotal in promoting inclusion and facilitating the inclusion of individuals with disabilities into society. Some studies [14,44,45] illuminate the inherent benefits of inclusive judo programs. In these settings, individuals with neurodevelopmental disorders and typically-developing (TD) peers derive advantages, fostering social interactions, growth, and overall well-being. In our review, the studies of Nanou et al. [44] and Lavisse [45] have been excluded because they did not have the methodological quality required in our inclusion criteria, but, as for Tomey [14], their results are very promising, justifying the use of an inclusive context and focus on this direction. In general, there is also a compelling need for longitudinal studies that can provide insights into the long-term impacts of judo. Research should especially focus on diverse neurodevelopmental conditions beyond ASD to fill the existing gap and enhance the inclusivity of judo programs. Furthermore, since it has already been studied for physical education teachers [46], investigating judo instructors’ perceptions and attitudes towards inclusivity could provide essential insights into these interventions’ practical challenges and opportunities. Additionally, participants’, stake-holders’, parents’, and judo peers’ perceptions must be included in the research to understand the challenges and strategies for practicing judo in an inclusive context.

## 5. Conclusions

This systematic literature review substantiates the multifaceted benefits of judo for individuals with NDDs, highlighting significant improvements across physical, social, emotional, and cognitive dimensions. The practice of judo, anchored in its foundational philosophies of optimal energy use and mutual prosperity, offers a unique blend of physical rigor and psychological reinforcement that is especially beneficial for this population. Judo promotes physical fitness and enhances social interactions and emotional well-being, making it a compelling intervention for individuals with NDDs. Looking forward, the expansion of judo programs into more inclusive settings appears both promising and necessary. Such environments can foster not only skill development but also social inclusion, offering benefits to both individuals with NDDs and their typically-developing peers. Additionally, future research should aim to explore the long-term impacts of judo, diversify the conditions studied, and employ more rigorous experimental designs to enhance the understanding and applicability of judo as a therapeutic intervention. In doing so, judo could become a widely endorsed practice within therapeutic and educational frameworks, promoting inclusivity and well-being among persons with neurodevelopmental challenges.

## Figures and Tables

**Table 1:** PICOTS framework synthesis of the results.

Authors (Study Quality Value %)	Population	Intervention Details	Comparator	Outcome Measures and Tools	Findings (with Symbols)
**ASD (13 Studies)**					
Tomey [14] (84%)	Five children with ASD and five typically-developed children (8–11 years)	5-week program, bi-weekly 50 min judo sessions, led by a certified coach. Inclusive setting.	None	Physical activity self-efficacy, enjoyment, barriers (pre/post). Focus groups, staff interviews.	↔ No significant differences in quantitative measures. Participants found the program enjoyable; staff noted ↑ in self-confidence and psycho-social health.
Burrell [15] (82%)	20 youth with ASD (8–17 years)	8-week program, weekly 45 min sessions, led by a certified coach.	None	Athletic self-competency, PA motivation, enjoyment (pre/post questionnaire).	↑ in psychosocial factors (not significant). Significant correlation (r = 0.43, *p* = 0.05) between PA motivation and attendance.
Rivera et al. [16] (86%)	25 youth with ASD (8–17 years)	8-week program, weekly 45 min sessions, led by a certified coach.	None	Behavior, lifestyle (Actigraph GT9X, ABC survey pre/post).	↑ Physical activity and sleep duration; ↔ slight improvement in sleep efficiency; ↓ ABC scores linked to ↑ attendance.
Renziehausen et al. [17] (90%	20 youth with ASD (children and adolescents)	10-week program, sessions once or twice per week, 45 min each, led by experienced coach.	None	Stress reactions, cortisol levels (pre/post surveys, cortisol measurements).	↓ Trends in stress subscales; ↔ cortisol levels no significant changes.
Garcia et al. [18] (87%)	14 youth with ASD (8–17 years)	8-week program, weekly 45 min sessions, led by 2 certified coaches.	None	MVPA and SB (Sedentary Behavior) (objective measures, descriptive statistics).	↑ MVPA; ↓ SB; positive insights into continued participation.
Rivera et al. [19] (91%)	25 youth with ASD (8–17 years)	8-week program, weekly 45 min sessions, led by certified instructor and additional staff.	None	Behavioral effects (ABC scores, parent interviews).	↔ ABC scores; 78% of parents noted ↑ in social skills and self-esteem.
Couto [20] (86%)	10 youth with ASD (6–14 years)	6-month program, bi-weekly 60-min sessions. judo group vs. control in school physical education.	Control group	Motor coordination (KTK test, student *t*-tests).	↑ Dynamic balance and monopedal jump in judo group; ↔ other metrics.
Morales et al. [21] (91%)	11 youth with ASD (9–13 years), IQ 60–70	8-week program, weekly 75 min sessions, led by 2 certified teachers with volunteer support.	None	GARS assessed in 4 stages.	↑ Improvement in all measured behaviors vs. baseline; ↓ post-lockdown.
Garcia et al. [22] (90%)	Nine adolescents with ASD (16.87 ± 1.36 years), 89% male	13.5-week program transitioning to remote due to COVID, initially in-person.	None	Program viability and practicality (survey, interviews).	92% attendance in remote sessions; overall satisfaction with remote judo.
Garcia et al. [23] (93%)	Nine youth with ASD, their parents, and eight youth without parents	15-week family-based vs. child-centric judo program, weekly 45 min sessions.	Child-centric group	Program feasibility (attendance, open-ended parent survey).	↑ Attendance and positive feedback in family program; ↑ physical gains emphasized in child-only group.
Morales et al. [24] (93%)	40 youth with ASD, 21 in program, 19 control, IQ 55–70	6-month program, weekly 90 min sessions, supported by volunteers.	Control group	Motor skills, psychosocial behaviors (TGMD-3, GARS-3, MANOCA).	↑ Significant improvements in motor skills and ↓ autism severity correlation.
Pierantozzi et al. [25] (93%)	40 youth with ASD (11.07 ± 1.73 years), IQ 55–70	6-month program, weekly 90 min sessions led by 2 certified judo teachers with volunteer support.	Control group	Health-related fitness (ALPHA-fitness battery, VO_2max_).	↑ Cardio-metabolic well-being and cardiorespiratory fitness. ↔ Reliability of peak effort tests.
Lockard, et al. [26] (86%)	24 youth, 15 males and 9 females (4–23 years), various developmental disabilities	Average of 2 years of participation.	None	Behavioral and social skills (Study Questionnaire, SSIS-SEL).	↑ Improvements in all behavioral and social categories assessed by parents.
IDD (2 studies)					
Zamanillo [27] (86%)	17 adults (18–49 years) with Intellectual Disabilities	5 years of adapted judo program	None	Adaptive behavior, quality of life (psychometric tests, narrative analysis).	↑ Overall well-being and self-esteem linked to ↑ adaptive skills.
Boguszewski et al. [28] (82%)	73 children (11.7 ± 2.6 years), majority with mild IDD	Undisclosed duration, frequency of judo classes not specified.	None	Physical fitness, social skills (parental survey post-intervention).	↑ Communication, confidence, lifestyle, socialization; ↔ pronunciation and word choice.
ADHD (1 study)					
Ludyga et al. [29] (91%)	57 children (8–12 years) with ADHD	3-month program, weekly 120 min judo sessions, alongside pharmacological treatment.	Wait-list control	Working memory, motor skills (Change Detection task, MABC-2, EEG).	↑ Working memory processes and visuospatial storage; ↔ motor skills.

Abbreviations: PA (Physical Activity), MVPA (Moderate to Vigorous Physical Activity), SB (Sedentary Behavior), IQ (Intelligence Quotient).

**Table 2:** Synthesis of the results.

Physical Findings	Social Findings	Emotional Findings	Cognitive Findings
Quantitative Findings			
Rivera [16]: ↑ Physical activity and sleep duration Garcia et al. [18]: ↑ MVPA; ↓ SB. Couto [20]: ↑ Dynamic balance and monopedal jump. Pierantozzi et al. [25]: ↑ Cardio-metabolic well-being and cardiorespiratory fitness Morales et al. [24]: ↑ Significant improvements in motor skills.	Garcia et al. [22] high attendance (92%) Lockard et al. [26]: ↑ Improvements in all behavioral and social categories. Boguszewski et al. [28]: ↑ Communication, lifestyle, socialization. Morales et al. [21]: ↑ Improvement in all measured behaviors vs. Baseline (GARS subscale)	Boguszewski et al. [28]: ↑, confidence Burrell [15]: ↑ in psychosocial factors. (not significant) Renziehausen et al. [17]: ↓ Trends in stress subscales. Rivera [16] ↓ ABC scores linked to ↑ attendance	Ludyga et al. [29]: ↑ Working memory processes and visuospatial storage. Burrell [15]: Significant correlation between PA motivation and attendance. Morales et al. [21]: ↑ Improvement in all measured behaviors vs. Baseline (GARS subscale)
Qualitative findings			
Garcia et al. [23]: ↑ Physical gains emphasized in child-only group.	Tomey [14]: ↑ psycho-social health. Rivera et al. [19]: 78% of parents noted ↑ in social skills Garcia et al. [22] overall satisfaction with remote judo. Garcia et al. [23]: Positive feedback in family program.	Tomey [14]: ↑ Self-confidence and participants found the program enjoyable Rivera et al. [19]: 78% of parents noted ↑ in self-esteem Zamanillo [27]: ↑ Overall well-being and self-esteem.	Zamanillo [27] ↑ adaptive skills

## Data Availability

All data generated or analyzed during this study are included in the article as Table(s).
